# Preparation of Layer-by-Layer Films Composed of Polysaccharides and Poly(Amidoamine) Dendrimer Bearing Phenylboronic Acid and Their pH- and Sugar-Dependent Stability

**DOI:** 10.3390/ma9060425

**Published:** 2016-05-28

**Authors:** Kentaro Yoshida, Keisuke Suwa, Jun-ichi Anzai

**Affiliations:** 1 School of Pharmaceutical Sciences, Ohu University, 31-1 Misumido, Tomita-machi, Koriyama, Fukushima 963-8611, Japan; k-yoshida@pha.ohu-u.ac.jp; 2 Graduate School of Pharmaceutical Sciences, Tohoku University, Aramaki, Aoba-ku, Sendai 980-8578, Japan; b4ym1017@s.tohoku.ac.jp

**Keywords:** layer-by-layer film, pH-sensitive, sugar-sensitive, dendrimer, phenylboronic acid, polysaccharide, alginic acid, carboxymethylcellulose

## Abstract

Layer-by-layer films composed of polysaccharides and poly(amidoamine) dendrimer bearing phenylboronic acid (PBA-PAMAM) were prepared to study the deposition behavior of the films and their stability in buffer solutions and in sugar solutions. Alginic acid (AGA) and carboxymethylcellulose (CMC) were employed as counter-polymers in constructing LbL films. AGA/PBA-PAMAM films were successfully prepared at pH 6.0–9.0, whereas the preparation of CMC/PBA-PAMAM film was unsuccessful at pH 8.0 and 9.0. The results show that the LbL films formed mainly through electrostatic affinity between PBA-PAMAM and polysaccharides, while, for AGA/PBA-PAMAM films, the participation of boronate ester bonds in the films was suggested. AGA/PBA-PAMAM films were stable in the solutions of pH 6.0–9.0. In contrast, CMC/PBA-PAMAM films decomposed at pH 7.5–9.0. The AGA/PBA-PAMAM films decomposed in response to 5–30 mM fructose at pH 7.5, while the films were stable in glucose solutions. Thus, AGA is useful as a counter-polymer for constructing PBA-PAMAM films that are stable at physiological pH and decompose in response to fructose.

## 1. Introduction

Layer-by-layer (LbL) films have attracted much attention because of their potential use in separation and purification [1], sensors [2,3], drug delivery [4,5], and so forth. LbL films are prepared by an alternate deposition of synthetic polymers [6,7,8] and biopolymers including proteins [9,10,11], polysaccharides [12,13,14], and DNA [15] on the surfaces of solid substrates and nanoparticles. Synthetic dyes and nanomaterials have also been employed as components of LbL films [16,17].

**Figure 1:**
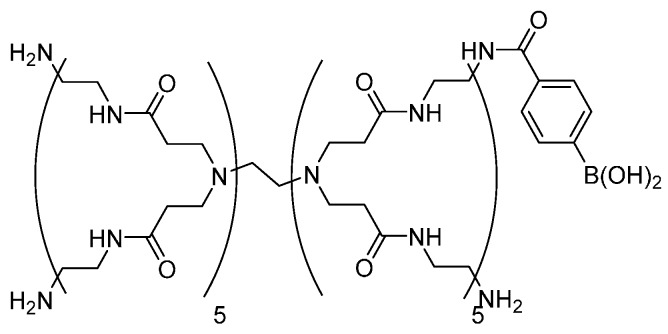
Chemical structure of PBA-PAMAM.

**Figure 2:**
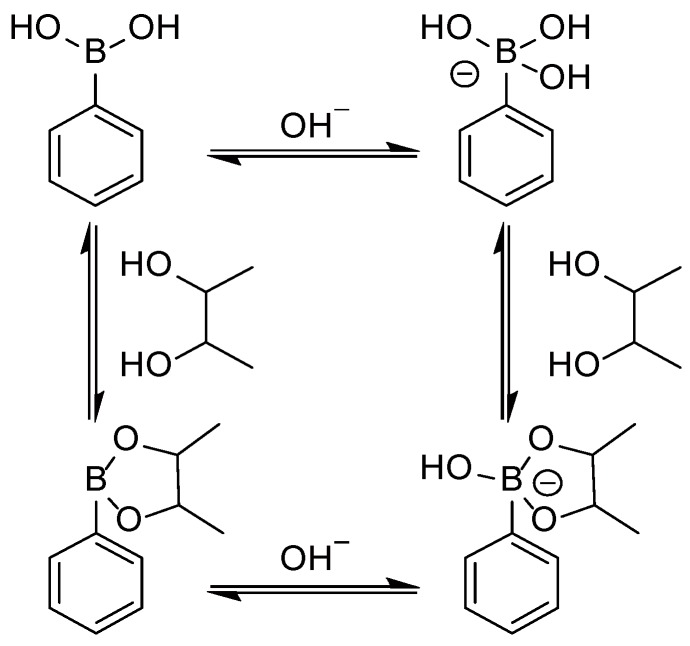
Binding equilibria of PBA to sugar and OH^−^ ion.

We have recently prepared stimuli-sensitive LbL films that decompose in response to sugars using phenylboronic acid (PBA)-bearing poly(amidoamine) dendrimers (PBA-PAMAM, Figure 1) for the applications of controlled release [18,19,20]. The LbL films were constructed by the alternate deposition of PBA-PAMAM and poly(vinyl alcohol) (PVA) [18,19] or glycoprotein (*i.e*., glucose oxidase, GOx) [20]. It is known that PBA binds diol compounds to form cyclic boronate ester bonds in aqueous media (Figure 2) [21]. Thus, PVA/PBA-PAMAM and GOx/PBA-PAMAM films were stabilized through boronate ester bonds between PBA-PAMAM and diol units in PVA or saccharide chains in GOx, respectively. In fact, PVA/PBA-PAMAM films were fairly stable in the solutions of basic pHs, whereas the films decomposed at weakly acidic pH [19]. On the other hand, GOx/PBA-PAMAM films decomposed to some extent, even at pH 7.0–8.0, while the films were stable in the solutions with pH higher than 8.5 [20]. These results show that pH stability of PBA-PAMAM–containing LbL films depends on the type of counter-polymers used. Other papers also reported pH-dependent stability of LbL films cross-linked with boronate ester bonds [18,22,23,24]. From the viewpoint of biomedical applications of LbL films, it is a prerequisite for the films to be stable under physiological conditions.

In this work, we used anionic polysaccharides, alginic acid (AGA) and carboxymethylcellulose (CMC) as counter-polymers for constructing AGA/PBA-PAMAM and CMC/PBA-PAMAM films to improve the pH stability as well as sugar response of the films. The LbL films would be stabilized through electrostatic bonds between positively charged PBA-PAMAM and the anionic polysaccharides. In addition, polysaccharides may form boronate ester linkages with PBA-PAMAM in the films because monomer units of the polysaccharides contain 1,2-diol residues. In fact, we have found that AGA/PBA-PAMAM films are stable at a physiological pH, though CMC/PBA-PAMAM films are unstable at pH 7.5 or higher. The present paper reports the preparation of LbL films composed of PBA-PAMAM and AGA or CMC and their pH stability as well as sugar response.

## 2. Materials and Methods

### 2.1. Materials

PAMAM dendrimer (4th generation, ethylenediamine core, 10% methanol solution) was purchased from Sigma-Aldrich Co. (St. Louis, MO, USA). AGA and CMC were obtained from Funakoshi Co. (Tokyo, Japan) and Tokyo Kasei Co. (Tokyo, Japan), respectively, and used without further purification. 4-Carboxyphenylboronic acid was obtained from Tokyo Kasei Co. 1-Ethyl-3-(3-dimethylaminopropyl)carbodiimide hydrochloride (EDC) and *N*-hydroxysuccineimide (NHS) were obtained from Nacalai Tesque Co. (Kyoto, Japan).

### 2.2. Preparation of PBA-PAMAM

PBA-PAMAM was prepared as reportedly [18,19]. Briefly, NHS ester of 4-carboxyphenylboronic acid was prepared by coupling NHS and 4-carboxyphenylboronic acid in *N*,*N*-dimethylformamide (Wako Pure Chemicals Industries, Ltd., Osaka, Japan) in the presence of EDC. The reaction mixture was stirred for 1 h at 0 °C and then for 12 h at room temperature. The crude product was purified by silica gel column chromatography (chloroform/methanol/acetic acid (Wako Pure Chemicals Industries, Ltd., Osaka, Japan), 100:10:1). The NHS ester was reacted with PAMAM in water for 12 h at room temperature. PBA-PAMAM was purified by dialysis in water using Spectra/Por^®^6 dialysis membrane (molecular weight cut-off: 3500, Spectrum Lab. Inc., Rancho Dominguez, CA, USA). Thus, PBA-PAMAM samples in which PBA residues were attached to 19 mol% and 39 mol% of the primary amino groups (referred to hereafter as 19%PBA-PAMAM and 39%PBA-PAMAM, respectively), as determined by the UV absorption spectra, were obtained.

### 2.3. Preparation of LbL Films

LbL films were prepared on the surface of a quartz slide (50 × 9 × 1 mm^3^) (Yazawa Co., Sendai, Japan), which had been cleaned in a mixture of sulfuric and chromic acids (Wako Pure Chemicals Industries, Ltd., Osaka, Japan). The quartz slide was alternately immersed in a 0.1 mg·mL^−1^ AGA or CMC solution and a 0.1 mg·mL^−1^ PBA-PAMAM solution for 20 min to deposit AGA/PBA-PAMAM or CMC/PBA-PAMAM films on the surface of the quartz slide. The PBA-PAMAM and polysaccharides solutions were prepared using 10 mM *N*-cyclohexyl-2-aminoethanesulfonate (CHES) buffer at pH 9.0, 10 mM 1-[4-(2-hydroxyethyl)-1-piperadinyl]ethanesulfonic acid] (HEPES) buffer at pH 7.0–8.0, and 10 mM 2-(*N*-morpholino)ethanesulfonic acid (MES) buffer at pH 6.0. All buffer solutions (Nacalai Co., Kyoto, Japan) contained 150 mM NaCl (Wako Pure Chemicals Industries, Ltd., Osaka, Japan). The quartz slide was rinsed in the working buffer for 5 min twice after each deposition. UV absorption spectra of the film-coated quartz slide were recorded in the working buffer on a UV-visible absorption spectroscope (UV-3100PC, Shimadzu, Kyoto, Japan) after each deposition of PBA-PAMAM and AGA or CMC.

### 2.4. pH-Dependent Stability

LbL films were deposited on one side of the quartz slide at pH 6.0. The slide was placed in a quartz cuvette (optical path length, 10 mm) filled with a buffer solution. The slide was placed near the sidewall of the cuvette, parallel to the light path, in order to avoid blocking the incident light. The absorbance of the buffer solution was monitored at 242 nm with gentle stirring to estimate the extent of film decomposition at different pHs. This protocol enables us to record absorption intensity of the buffer solution, not that of the films, in which decomposed film components are dissolved.

### 2.5. Sugar-Induced Decomposition

The sugar-induced decomposition of the films were studied by monitoring the absorbance of 10 mM HEPES buffer solution at pH 7.5, in which LbL film-coated quartz slide was immersed, upon successive addition of 1–30 mM glucose and fructose (Wako Pure Chemicals Industries, Ltd., Osaka, Japan). All measurements were carried out at room temperature (*ca.* 23 °C).

## 3. Results and Discussion

### 3.1. Preparation of LbL Film

**Figure 3:**
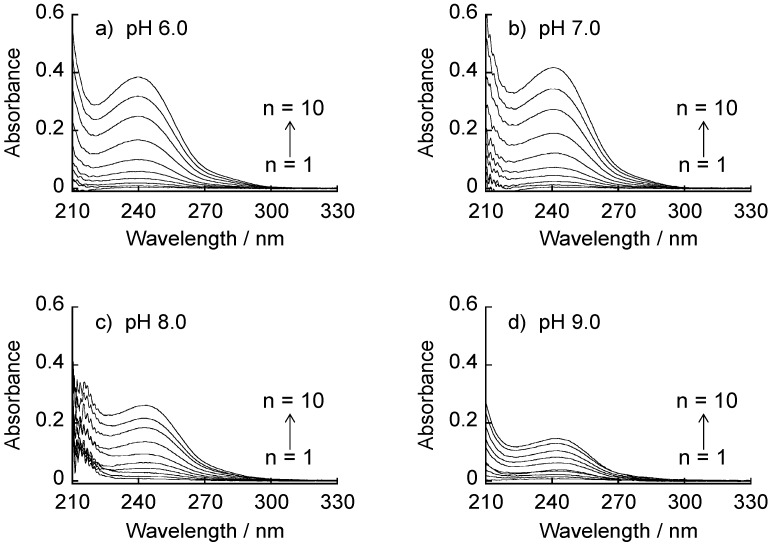
UV absorption spectra of (AGA/39%PBA-PAMAM)_n_ LbL films (*n* = 1–10) prepared at pH 6.0–9.0. (**a**) UV absorption spectra recorded at pH 6.0; (**b**) UV absorption spectra recorded at pH 7.0; (**c**) UV absorption spectra recorded at pH 8.0; (**d**) UV absorption spectra recorded at pH 9.0.

PBA-PAMAM may be built into LbL films because positively charged primary amino groups of PBA-PAMAM are available for the electrostatic binding to carboxylate residues of AGA and CMC. Figure 3 shows UV absorption spectra of (AGA/39%PBA-PAMAM)_n_ films (*n* = 1–10) prepared at pH 6.0, 7.0, 8.0 and 9.0. The absorption band of the films around 240 nm, which originates from 39%PBA-PAMAM, increased with increasing the number of AGA/39%PBA-PAMAM bilayers, showing that (AGA/39%PBA-PAMAM)_10_ LbL films were successfully constructed at pH 6.0–9.0. The intensities of the absorption bands of the films prepared at pH 6.0 and 7.0 were higher than those of the films deposited at pH 8.0 and 9.0, suggesting that electrostatic affinity between AGA and 39%PBA-PAMAM is a main driving force for the film formation. It is envisaged that the density of the positive charges in 39%PBA-PAMAM, which originates from the primary and tertiary amino groups, is pH-dependent in the range of pH 6.0–9.0. The p*K*_a_ values of primary and tertiary amino groups of PAMAM are reported to be 9.23 and 6.30, respectively [25]. Thus, the surface of 39%PBA-PAMAM should be highly protonated at pH 6.0 and 7.0, resulting in strong binding to AGA (p*K*_a_, 3.5–5.0 [26]) in the film. In contrast, the amount of positive charges is lower at pH 8.0 and 9.0, which limits electrostatic binding of 39%PBA-PAMAM to AGA. It is also plausible that the LbL films are stabilized in part by boronate ester bonds between 39%PBA-PAMAM and AGA. A possible participation of boronate ester bonds in the LbL films will be further discussed in the later section in relation to the preparation of CMC/39%PBA-PAMAM films and their pH stability.

**Figure 4:**
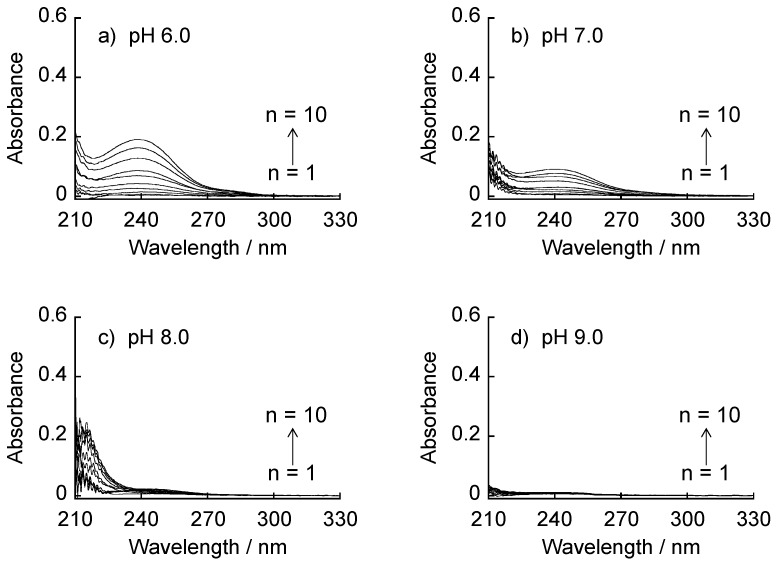
UV absorption spectra of (CMC/39%PBA-PAMAM)_n_ LbL films (*n* = 1–10) prepared at pH 6.0–9.0. (**a**) UV absorption spectra recorded at pH 6.0; (**b**) UV absorption spectra recorded at pH 7.0; (**c**) UV absorption spectra recorded at pH 8.0; (**d**) UV absorption spectra recorded at pH 9.0.

Figure 4 shows UV absorption spectra of (CMC/39%PBA-PAMAM)_n_ films (*n* = 1–10) prepared at pH 6.0–9.0. The absorption bands of the films increased with increasing the number of depositions at pH 6.0 and 7.0, while film deposition was unsuccessful at pH 8.0 and 9.0. The results suggest that the deposition of (CMC/39%PBA-PAMAM)_10_ films relies on the electrostatic force of attraction, as in the case for the (AGA/39%PBA-PAMAM)_10_ film. The deposition behavior of the (CMC/39%PBA-PAMAM)_10_ films is somewhat different from that of the (AGA/39%PBA-PAMAM)_10_ films. First, deposition of the (CMC/39%PBA-PAMAM)_10_ film was suppressed almost completely at pH 8.0 and 9.0, in contrast to the successful deposition of the (AGA/39%PBA-PAMAM)_10_ films even at the higher pHs. In addition, the intensities of the 240 nm band of the (CMC/39%PBA-PAMAM)_10_ films are always lower than those of the (AGA/39%PBA-PAMAM)_10_ films prepared at the same pH. Thus, a larger amount of 39%PBA-PAMAM was deposited in the (AGA/39%PBA-PAMAM)_10_ films than in the CMC-based films. These results cannot be fully rationalized only from the electrostatic binding between 39%PBA-PAMAM and the polysaccharides because the charge density (or the number of carboxylate residues) of AGA and CMC is nearly comparable. Therefore, the results suggest the participation of a secondary binding force, *i.e.*, boronate ester bonds, in the (AGA/39%PBA-PAMAM)_10_ films. AGA is a polysaccharide composed of d-mannuronic acid and l-gluronic acid, which are derived from d-mannose and l-glucose, respectively, while CMC consists of carboxymethylated d-glucose units. It has been reported that mannose polymer, *i.e.*, mannan, forms boronate ester bonds with PBA polymer to provide LbL films [27]. The binding constant of PBA to d-mannose (13 M^−^^1^ at pH 7.4) is higher than that to d-glucose (4.6 M^−1^ at pH 7.4) [21]. These data support that the (AGA/39%PBA-PAMAM)_10_ films were stabilized in part by boronate ester bonds, which was not the case for the (CMC/39%PBA-PAMAM)_10_ films.

In order to evaluate the effect of PBA content in PBA-PAMAM on the film formation, 19%PBA-PAMAM was used to construct LbL films. Figures S1 and S2 in Supplementary Materials show UV absorption spectra of (AGA/19%PBA-PAMAM)_n_ and (CMC/19%PBA-PAMAM)_n_ films (*n* = 1–10) prepared at pH 6.0–9.0. The deposition behavior of the films was similar to that of the 39%PBA-PAMAM films. However, absorption intensities in the UV spectra of the (AGA/19%PBA-PAMAM)_n_ and (CMC/19%PBA-PAMAM)_n_ films were always lower than those of 39%PBA-PAMAM films prepared at the same pH. This is reasonable because the absorption bands around 240 nm are arising from PBA residues in PBA-PAMAM. The loadings of PBA-PAMAM in the (AGA/19%PBA-PAMAM)_10_ and (AGA/39%PBA-PAMAM)_10_ films prepared at pH 6.0 are calculated to be 1.47 × 10^−9^ and 1.23 × 10^−9^ mole/cm^2^, respectively, showing that both the films contain comparable amounts of PBA-PAMAM. The loadings of PBA-PAMAM in the (CMC/19%PBA-PAMAM)_10_ and (CMC/39%PBA-PAMAM)_10_ films were prepared at pH 6.0 are 9.69 × 10^−10^ and 6.06 × 10^−10^ mole/cm^2^, respectively. Thus, the amount of PBA-PAMAM in the films did not significantly depend on the degree of PBA substitution in PBA-PAMAMs.

### 3.2. pH-Dependent Stability

It is reasonable to assume that PBA-PAMAM films are sensitive to pH because the amounts of positive charges of the PBA-PAMAM used are dependent on the pH of the media. The addition of an OH^−^ ion to the boron atom of the PBA moiety results in a tetragonal form, which has a stronger binding affinity than that of the parent trigonal form (Figure 2) [21]. In fact, we have found that PBA-PAMAM films decomposed in response to pH changes.

**Figure 5:**
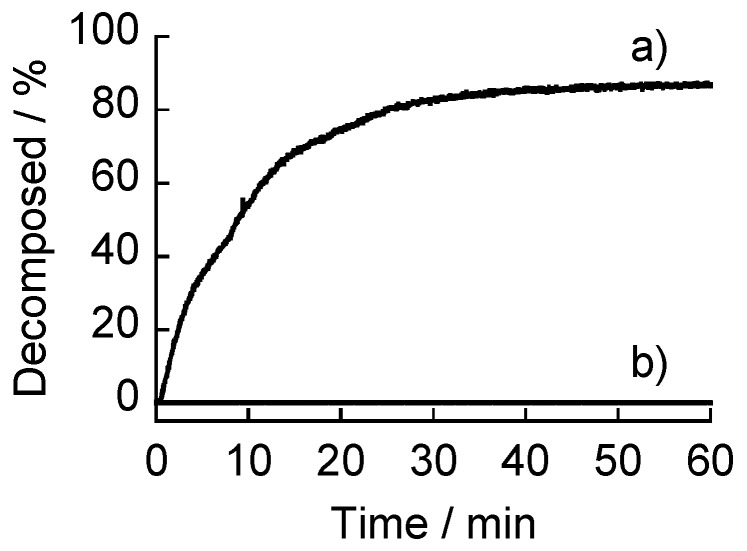
The kinetics of the decomposition of (CMC/39%PBA-PAMAM)_10_ (**a**) and (AGA/39%PBA-PAMAM)_10_ films (**b**) in buffer solution at pH 9.0.

**Figure 6:**
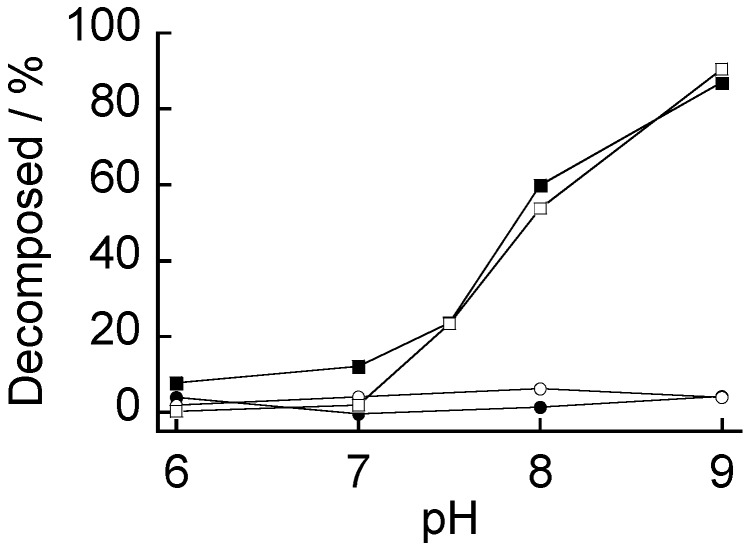
pH-dependent stability of (CMC/19%PBA-PAMAM)_10_ (□), (CMC/39%PBA-PAMAM)_10_ (■), (AGA/19%PBA-PAMAM)_10_ (○), and (AGA/39%PBA-PAMAM)_10_ films (●). The percentages of decomposition after immersing the films in the buffer solutions for two hours are plotted.

Figure 5 shows the decomposition kinetics of the (AGA/39%PBA-PAMAM)_10_ and (CMC/39%PBA-PAMAM)_10_ films in a buffer solution of pH 9.0. The (CMC/39%PBA-PAMAM)_10_ film decomposed in the basic solution, whereas the (AGA/39%PBA-PAMAM)_10_ film did not decompose at all. This shows that the pH stability of PBA-PAMAM films significantly depends on the type of polysaccharide used. In a separate experiment, the pH stability of the films was evaluated by immersing the films in solutions of pH 6.0–9.0 (Figure 6). The (AGA/19%PBA-PAMAM)_10_ and (AGA/39%PBA-PAMAM)_10_ films were highly stable in the solutions of pH 6.0–9.0, while the (CMC/19%PBA-PAMAM)_10_ and (CMC/39%PBA-PAMAM)_10_ films decomposed at pH 7.5–9.0. Both the films are electrostatically stabilized at pH 6.0–7.0 because the dendrimers contain higher amounts of positive charges. However, the amount of positive charges decreased at the higher pH, resulting in the loss of electrostatic affinity of the PBA-PAMAM to CMC. On the other hand, the AGA-based films were stable even at the higher pH owing to the boronate ester bonds formed between PBA-PAMAM and AGA.

LbL films based on boronate ester bonds are often unstable in neutral and acidic solutions because boronate ester bonds are stable only when the boron atom assumes a negatively charged tetragonal form in basic media. For instance, LbL films consisting of PBA-modified poly(acrylic acid) and mannan were stable at pH 9.0 whereas the films decomposed at pH 8.0 or lower [27]. In addition, LbL films composed of 39%PBA-PAMAM and PVA decomposed at pH 7.0 or lower [19]. In this regard, the present results demonstrate that use of anionic polysaccharides, such as AGA and CMC, is effective for stabilizing PBA-PAMAM LbL films in neutral and weakly acidic media.

In this context, it is noteworthy that polyelectrolyte multilayer films can be stabilized by non-electrostatic interactions, such as hydrogen bonds, in spite of charge imbalance between the components [28,29,30].

### 3.3. Sugar-Induced Decomposition

Sugar-sensitive LbL films have been widely studied for the development of biosensors and delivery systems [22,23,24,31,32,33,34,35]. LbL films that decompose in response to sugars but are stable under physiological conditions would be highly useful for biomedical applications. PBA-PAMAM films may be destabilized or decomposed when the films are exposed to sugars as a result of the addition of sugars to PBA-PAMAM. We have evaluated the sugar response of the (AGA/19%PBA-PAMAM)_10_ and (AGA/39%PBA-PAMAM)_10_ films because these films are stable at physiological pH (see Figure 6). In fact, we have found that these films decomposed in part in fructose solutions.

**Figure 7:**
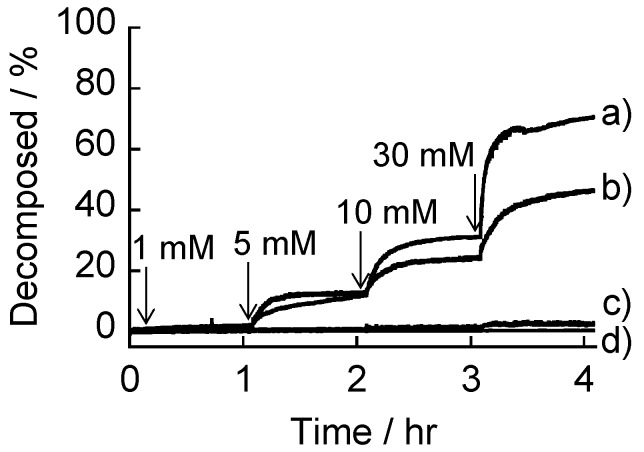
Decomposition of (AGA/19%PBA-PAMAM)_10_ (**b**,**c**) and (AGA/39%PBA-PAMAM)_10_ films (**a**,**d**) upon successive addition of fructose (**a**,**b**) and glucose (**c**,**d**) in buffer solutions at pH 7.5. LbL films were prepared at pH 7.5.

Figure 7 shows the decomposition behavior of the (AGA/19%PBA-PAMAM)_10_ and (AGA/39%PBA-PAMAM)_10_ films in glucose and fructose solutions at pH 7.5. Both the films decomposed upon the addition of 5–30 mM fructose, depending on the concentration of fructose. Two different mechanisms may be involved in the film decomposition. First, boronate ester bonds between PBA-PAMAM and AGA in the films were cleaved by competitive binding of fructose to PBA-PAMAM. It is reasonable to assume that some portions of PBA residues in the films are involved in boronate ester bonds with AGA at pH 7.5 whereas others are free from ester bonds. Second, the binding of fructose to free PBA residues enhanced the amount of negative charges of PBA-PAMAM because the p*K*_a_ of boronate esters is usually more acidic than that of free PBA (see Figure 2). The negatively charged PBA-PAMAM induced destabilization of the films owing to the electrostatic repulsion by AGA. In both mechanisms, the formation of boronate ester bonds between PBA-PAMAM and added fructose is responsible for the film decomposition. Thus, it is quite natural that the (AGA/39%PBA-PAMAM)_10_ film was more sensitive to fructose than the film made of 19%PBA-PAMAM. On the other hand, the binding affinity of glucose to PBA-PAMAM was too low at pH 7.5 to induce film decomposition. In other words, the (AGA/19%PBA-PAMAM)_10_ and (AGA/39%PBA-PAMAM)_10_ films can be decomposed selectively by the addition of fructose.

## 4. Conclusions

LbL films comprising PBA-PAMAM and polysaccharides were successfully prepared by the alternate deposition of PBA-PAMAM and AGA or CMC on the surface of a quartz slide. The LbL films were stabilized mainly by the electrostatic affinity between PBA-PAMAM and polysaccharides at pH 6.0–7.0 while boronate ester bonds were involved in the AGA/PBA-PAMAM films at pH 8.0–9.0. Thus, the AGA/PBA-PAMAM films were stable in the range of pH 6.0–9.0. The AGA/PBA-PAMAM films decomposed in the presence of 5–30 mM fructose at pH 7.5, whereas glucose did not induce decomposition of the films. The results showed the usefulness of AGA as a counter-polymer in constructing sugar-sensitive PBA-PAMAM films that are stable at physiological pH.

## Supplementary Materials

The following are available online at www.mdpi.com/1996-1944/9/6/425/s1.
