# Supplementary material for: Preparation of Layer-by-Layer Films Composed of Polysaccharides and Poly(Amidoamine) Dendrimer Bearing Phenylboronic Acid and Their pH- and Sugar-Dependent Stability
Source: Materials (Basel). 2016 May 28;9(6):425. doi: 10.3390/ma9060425 (PMC5456809; doi:10.3390/ma9060425)
Supplement: Supplementary file 1 [file materials-09-00425-s001.docx]

<Supporting Materials>

Preparation of Layer-By-Layer Films Composed of Polysaccharides and Poly(Amidoamine) Dendrimer Bearing Phenylboronic Acid And Their pH- and Sugar-Dependent Stability

Kentaro Yoshida, Keisuke Suwa, and Jun-ichi Anzai

**Figure S1.** UV spectra of (AGA/19%PBA-PAMAM)_n_ films (n = 1–10) prepared at pH 6.0–9.0.

**Figure S2.** UV spectra of (CMC/19%PBA-PAMAM)_n_ films (n = 1–10) prepared at pH 6.0–9.0.
